# Void Distribution in Zeolite Warm Mix Asphalt Mixture Based on X-ray Computed Tomography

**DOI:** 10.3390/ma12121888

**Published:** 2019-06-12

**Authors:** Junda Ren, Chao Xing, Yiqiu Tan, Nan Liu, Jingyi Liu, Liying Yang

**Affiliations:** 1 Liaoning Transportation Research Institute Co. Ltd., Key Laboratory of Transport Industry of Expressway Maintenance Technology, Shenyang 110000, China; renjunda89@163.com (J.R.); liunan0613@163.com (N.L.); jingyiliu007@163.com (J.L.); 2 School of Transportation Science and Engineering, Harbin Institute of Technology, Harbin 150000, China; 3 School of Transportation Science and Engineering, State Key Laboratory of Urban Water Resource and Environment, Harbin Institute of Technology, Harbin 150000, China; tanyiqiu@hit.edu.cn; 4 Beijing Municipal Road & Bridge Building Material Group Co. Ltd., Beijing 100000, China; yangliying_q@sina.com

**Keywords:** asphalt mixture, warm mix, zeolite, CT method, void distribution

## Abstract

Warm mix asphalt mixtures have the advantages of energy saving, emission reduction and good road performance. Zeolite asphalt mixtures, as a warm mixing technology, have been applied in the world. To understand the warm mix mechanism of zeolite warm mix asphalt mixture, the mesoscale structure of zeolite asphalt is studied. Micro computed tomography (CT) is utilized to obtain the internal structure image of zeolite-modified asphalt and asphalt mixture. The quantity and volume of voids are used as internal void distribution evaluation indexes. The results indicate that with respect to the void distribution in zeolite-modified asphalt, with the increase of temperature, there is an obvious evolution trend of smaller voids to larger voids. With respect to the voids in the zeolite-modified asphalt mixture, the zeolite asphalt mixture is equivalent to hot mix asphalt mixture when it is above 120 °C, while below 120 °C, the maximum and average void volumes increase significantly, making it difficult for the mixture to achieve ideal compaction effect.

## 1. Introduction

Warm mix asphalt mixture can effectively reduce mixing and construction temperature, thereby reducing energy consumption, asphalt aging and emission of harmful gas in the mixing and construction process. Under the premise of ensuring mechanical performance and durability, it realizes green and economic construction technology of asphalt pavement. Therefore, the technology is widely used around the world, and has become a hot research topic [1,2,3]. Warm mix additives include organic and chemical elements, as well as zeolites, all of which change the physical properties of asphalt. Specifically, they first reduce the viscosity of the binder, and lower the mixing temperature by 10–30 °C [4]. Topal [5,6,7] compared the performance of warm mixtures with organic, chemical, natural zeolite and synthetic zeolite, and obtained the optimal additional amounts. Zeolite, as a kind of warm mix additive, is dependent on the tiny foams produced by evaporation or by the boiling of crystal water in the zeolite. These tiny foams play a role as a lubricant in the mixing process, so as to improve the workability and compaction performance of the mixture. Viscosity is an important technical index, affecting the mixing of the asphalt mixture. Previous studies have shown that the decrease of construction temperature of zeolite warm mix asphalt mixture is mainly due to the change in asphalt viscosity due to zeolite. Compared with normal asphalt, the viscosity of zeolite-modified asphalt is lower. However, in recent years, it has been demonstrated that zeolite cannot reduce the viscosity of asphalt. Zeolite plays a warm mix role mainly through the lubrication of asphalt and aggregate by releasing water. Akisetty [8] investigated the influence of zeolite on asphalt properties, and it was found that the addition of zeolite increased the viscosity of the asphalt mortar. Wasiuddin [9] tested the viscosity of zeolite asphalt mortar using a rotating viscometer, and it was found that zeolite did not play a role in reducing the viscosity. Therefore, it was concluded that the viscosity–temperature curve was not able to reveal the warm mixing mechanism of zeolite additives. Lee [10] studied the performance of asphalt blended with zeolite and organic viscosity reducer. It was found that the viscosity of asphalt blended with zeolite increased, while organic products effectively reduced the viscosity of the asphalt. Adding zeolite instead of mineral powder into the asphalt mixture and lowering the mixing temperature will inevitably have a certain impact on the performance of the asphalt mixture. Hurley [11] studied the performance of zeolite warm mix asphalt mixture through indoor tests and field tests. It was proved that the zeolite reduces the construction temperature by about 20 °C during the paving process, and it had no adverse effect on the road performance. Visscher [12] showed that using natural zeolite as a warm mix agent could reduce the mixing temperature, but that this would affect the water stability of asphalt mixture. Woszuk [13,14] investigated the effect of zeolite properties on asphalt foaming, and the results showed that the foaming effect was strongly dependent on the amount of water in the zeolite structure. The warm mix performance of zeolite is affected by tiny foams, which are produced by the evaporation or boiling of crystal water in the zeolite. Therefore, the micro voids property is important for revealing the warm mix mechanism. In recent years, Computed tomography has been widely used in the microstructure investigation of asphalt mixtures [15,16,17]. For the investigation of voids in asphalt mixtures, Masad [18] studied the void distribution in the vertical direction of the asphalt mixture. Tashman [19] compared the voids property of asphalt mixture with different compaction methods. The relation between void distribution and the performance of asphalt mixtures was also investigated [20,21,22,23]. This has been demonstrated to be effective in the characterization of void distribution. Thus, in order to understand the warm mix mechanism of zeolite warm mix asphalt mixture, this study investigates the void distribution in asphalt and asphalt mixture based on CT technology.

## 2. Materials and Methods

AC-20 asphalt mixture is selected for investigating the void properties. The binder used in this paper is grade 70 pen bitumen, and the coarse and fine aggregates are from Beijing limestone. The properties of asphalt and aggregates are presented in Table 1, Table 2 and Table 3. Zeolites are porous, hydrated aluminosilicates with a general formula of M_x/m_[(AlO_2_)_x_(SiO_2y_)]. H_2_O, where M_x/m_ designates ion-exchangeable cations, and the [(AlO_2_)_x_(SiO_2y_)] unit constitutes the zeolite framework [24,25]. Commercial zeolite from PR INDUSTRIE (PRI) is used for the warm mix additive, and the addition amount is 0.3% by weight of the asphalt mixture. The properties of zeolite are shown in Table 4. 

Based on the gradation limitation of Chinese specification JTG F40-2004 [26], the gradation of the AC-20 hot mix asphalt mixture is given in Table 5. According to the Marshall design method, the optimum asphalt–aggregate ratio is 4.4%, and the corresponding void ratio is 4.4%. To compare the difference between hot mix and warm mix asphalt mixture, the zeolite asphalt mixture adopts the same gradation and asphalt–aggregate ratio as the hot mix asphalt mixture.

For describing the properties of the voids, the following two problems should be solved: the first is the identification of voids according to the difference between asphalt, aggregates and voids; the second is the reconstruction and quantification of the distribution of voids. In view of these two problems, CT technology, as a nondestructive testing method, provides and approach for extracting the characteristics of voids in the asphalt mixture. For many years, it has been widely used in the medical field. In recent years, CT technology has been gradually applied in the analysis of the internal structure of asphalt mixtures. In combination with a digital imaging process, it allows the distribution of asphalt, aggregates and voids to be identified. Phoenix V | tome | x s micro CT from Harbin Institute of Technology is utilized for microstructure detection. 

**Figure 1:**
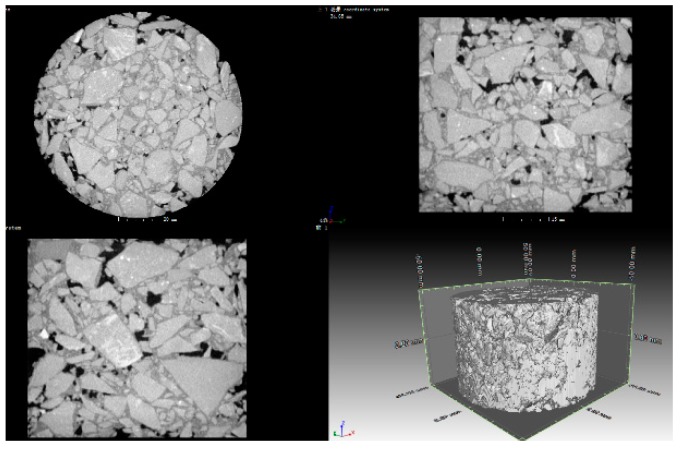
3D vision of asphalt mixture sample.

The principle of CT scanning is that X-rays are emitted from a radiation source, which can penetrate the object. Different materials can absorb different intensities of radiation, and the detector receives attenuated X-ray information to recognize the different materials. After CT scanning, the datos|x reconstruction software is used to reconstruct the three-dimensional volume data according to the ray attenuation, and extract all the inner and outer surfaces of the object from the volume data. Based on the reconstruction data, VG Studio MAX 2.2 is used to visualize the digital object, and extract the different components information. The 3D vision and three-view slice images are shown in Figure 1. 

## 3. Results

### 3.1. Void Distribution in Zeolite-Modified Asphalt 

**Figure 2:**
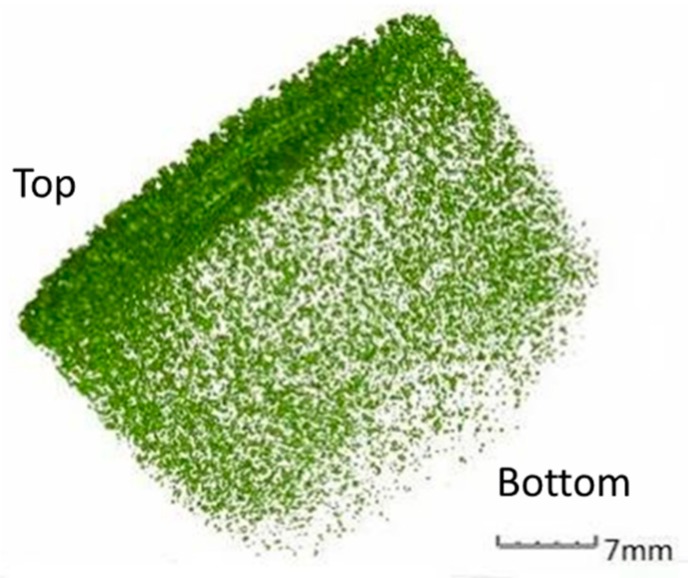
Three-dimensional distribution of voids in the asphalt.

For investigating the void distribution in asphalt binder, zeolite is added to the asphalt binder. The zeolite amount added is 0.3% by weight of the asphalt mixture, and the optimum asphalt–aggregate ratio is 4.4%. Therefore, the proportion of zeolite in the asphalt binder is 7.1%. The asphalt binder is heated to 120 °C and mixed with zeolite for 1 min. After mixing, the asphalt is poured into a cylindrical container. After cooling, the asphalt is scanned by CT equipment. Images are taken every 0.1 mm along the height of the specimen during scanning. After processing the scanned images, the quantity of the voids is counted, and the distribution of the voids in the asphalt is analyzed accordingly. The spatial distribution of voids in the asphalt is shown in Figure 2.

From Figure 2, it can be seen that after adding zeolite to the asphalt, the voids produced by the zeolite in the asphalt exhibit characteristics of “more and larger voids at the top”. That is to say, the voids in the asphalt collect at the surface of asphalt, and the diameter of the bubbles is larger; meanwhile, at the bottom of asphalt, the bubbles are fewer and smaller. The spatial distribution of voids generated by the zeolite is related to the content of zeolite water, the existence state of the zeolite water, and temperature [27]. To analyze the effect of temperature on the size and distribution of voids, zeolite is added to asphalt at 100 °C and 120 °C, and then the zeolite-modified asphalt is mixed uniformly, molded and cooled. After CT scanning and image processing, the distribution of the voids is analyzed. The quantity and volume of voids in the asphalt are shown in Table 6 and Table 7, respectively, and the unit of measurement for void volume is mm^3^.

From Table 6, it can be seen that at the condition of 100 °C, the voids system in zeolite asphalt is dominated by voids smaller than 0.1 mm^3^. The quantity of voids smaller than 0.1 mm^3^ is larger than at 120 °C, but the quantity percentage is lower. This phenomenon shows that with the increase of temperature, the evolution trend of smaller voids to larger voids is obvious. Void volume distribution can better explain the evolution of voids, as shown in Table 7. First of all, the voids ratio is much larger at 120 °C, showing that temperature has a significant effect on the overall voids ratio. For the voids volume distribution, the volume of micro voids smaller than 0.1 mm^3^ is greater, and the volume distribution of different sized voids is more uniform at 100 °C. However, for 120 °C, voids larger than 1 mm^3^ account for the vast majority. The results above illustrate that with the increase of temperature, the smaller voids (<1 mm^3^) are merged into larger voids (>1 mm^3^). Above all, the effect of temperature is reflected in increasing the overall void ratio and changing the distribution of different size voids, which include the decrease in void quantity and the increase in the volume of larger voids (>1 mm^3^).

### 3.2. Void Distribution of Asphalt Mixture with Zeolite Additive

**Figure 3:**
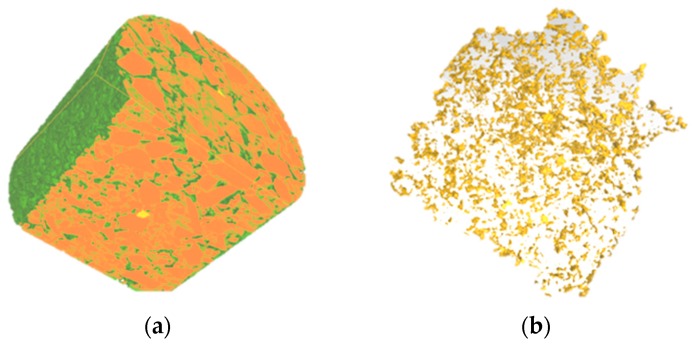
Digital sample and void of asphalt mixture. (**a**) Three-dimensional digital sample; (**b**) three-dimensional distribution of void.

The combination of industrial CT and image technology provides a way to study the internal structure of materials. The internal voids characteristics and distribution of zeolite warm mix asphalt mixture are investigated by CT scanning technology, which can directly evaluate the compaction effect. The images of zeolite asphalt mixture compacted at different temperatures are analyzed by CT scanning. Figure 3 shows the three-dimensional CT images of the asphalt mixture.

**Figure 4:**
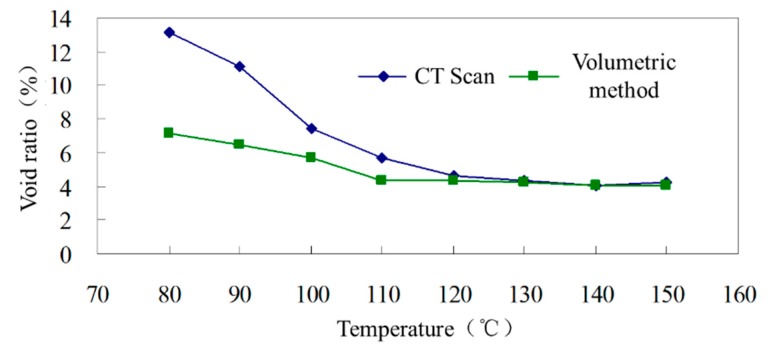
Change of void ratio at different compacting temperatures.

The internal voids of zeolite asphalt mixtures mixed and compacted at different temperatures are extracted and analyzed. Among them, the zeolite asphalt mixtures are mixed and compacted by Marshall method at 80–140 °C. In contrast, the temperature for the hot mix asphalt mixture is 150 °C. Before mixing, the aggregate and asphalt binder are heated at the same temperature for 4 h. The void ratio of each specimen is extracted and calculated by CT method, and compared with that calculated by volumetric method in Chinese specification JTG E20-2011 [28]. Based on the comparison between warm and hot mix asphalt mixture, the compaction effect of zeolite asphalt mixture can be evaluated. The voids of asphalt mixtures at different temperatures are shown in Table 8 and Figure 4.

In Figure 4, the void ratio of hot mix asphalt mixture at 150 °C is presented in the same figure for comparison. The void ratio of zeolite asphalt mixture decreases with the increase of compacting temperature, and the void ratio for hot and warm mix mixtures is similar at the temperature range between 120 °C and 150 °C. This illustrates that the compaction temperature of zeolite asphalt mixture should be maintained at above 120 °C. For the same asphalt mixture, the void ratio calculated by the volumetric method basically coincides with that calculated by CT scan above 120 °C. When the temperature is lower than 120 °C, the void ratio obtained by CT scan is larger than that obtained using the volumetric method. The lower the temperature is, the larger the difference between the two results.

The main reason for this difference is that in the test of the volumetric method, some large surface voids are not included in the void ratio calculation, because they do not absorb water. However, the CT scan method utilizes the material density difference to identify voids, and surface voids can be identified as voids. The difference between void recognition methods leads to the difference in results between the CT scan and volumetric methods. The lower the temperature is, the larger surface voids there are, and the greater the difference between the two void ratio results.

Void ratio can be used to evaluate the compaction effect of asphalt mixture macroscopically. However, as a composite three-dimensional structure, the void ratio cannot fully reflect its void structure. The distribution of internal voids of asphalt mixture with the same void ratio may be different. With the help of CT technology, the characteristics of void structure in asphalt mixture can be described at micro scale. For zeolite asphalt mixture, it can also be used as a method for evaluating compaction effect.

**Figure 5:**
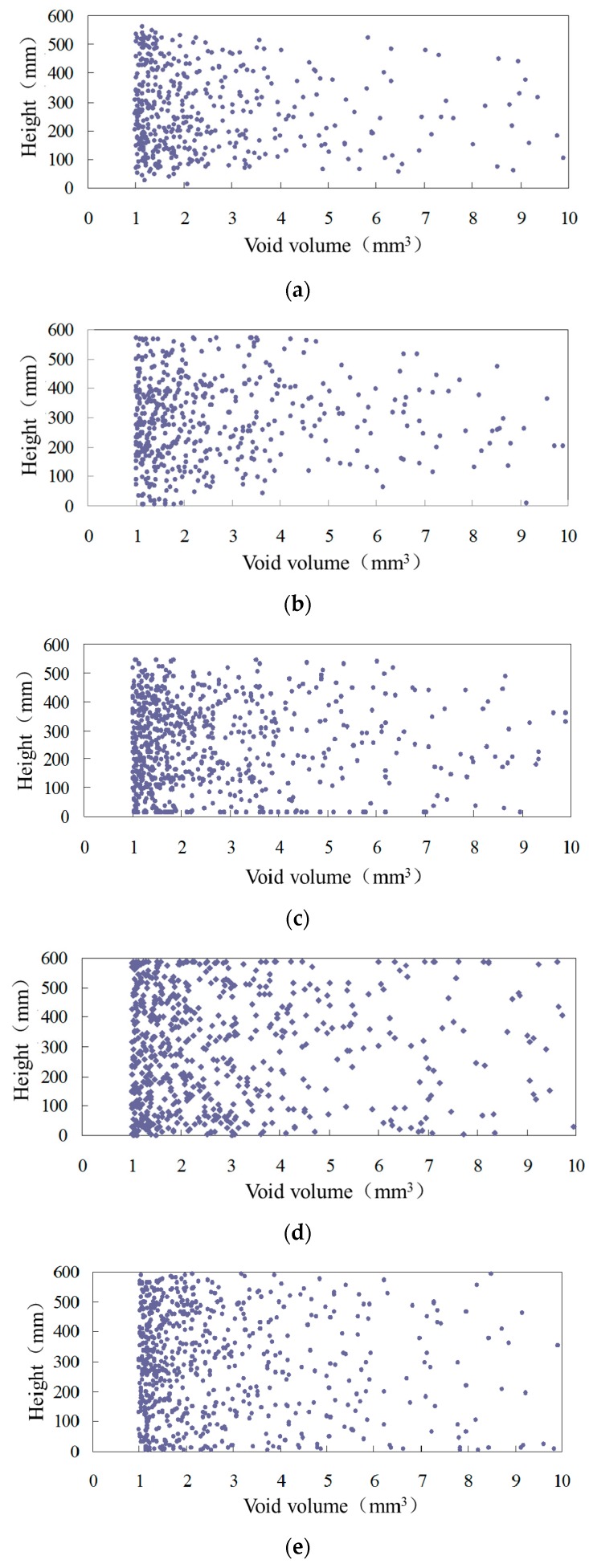
1–10 mm^3^ void distribution of asphalt mixture. (**a**) 80 °C; (**b**) 90 °C; (**c**) 120 °C; (**d**) 140 °C; (**e**) 150 °C.

**Figure 6:**
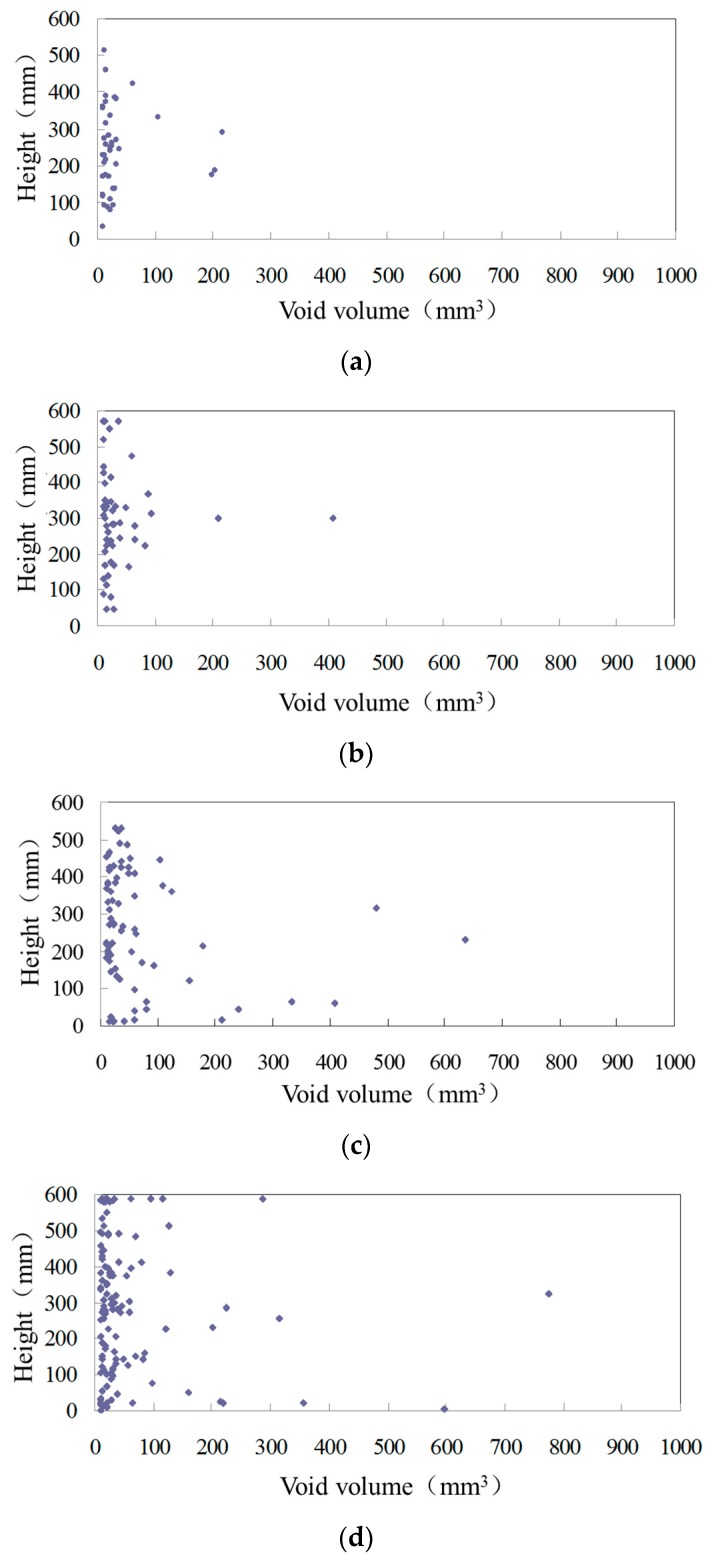

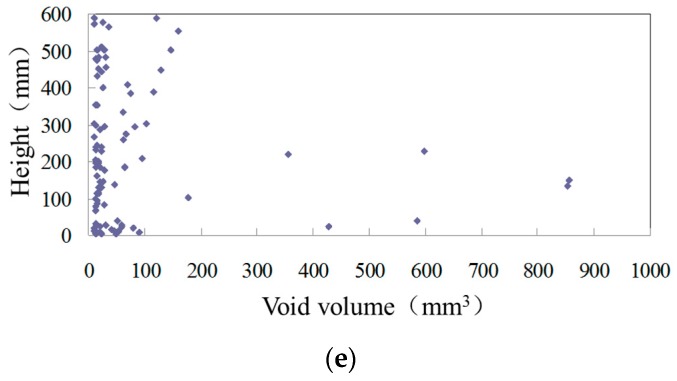
10–1000 mm^3^ void distribution of asphalt mixture. (**a**) 80 °C; (**b**) 90 °C; (**c**) 120 °C; (**d**) 140 °C; (**e**) 150 °C.

For the description of the spatial distribution of voids, the centroids of voids at different heights of the sample are extracted. To fully reflect the distribution of voids in the sample, the voids are divided into 1–10 mm^3^ and 10–1000 mm^3^ categories in order to draw the void distribution maps. The distributions of different sized voids are shown in Figure 5 and Figure 6, in which the longitudinal coordinates are the height of the specimen and the abscissa coordinates are the volume of the voids. Each point in the figure represents a void with a certain volume at a certain height of the specimen, that is, each point represents a void.

In the internal void distribution maps of Figure 5, voids with different volumes have different distribution densities. The smaller the void volume, the larger the density, and the more uniform the distribution along the vertical direction. Most of the voids in asphalt mixtures are between 1 mm^3^ and 2 mm^3^, and these have the widest distribution range and are densely distributed at different heights of specimens. This shows that the small voids are most widely distributed at different heights of asphalt mixtures. With the increase in void volume, the distribution density in the mixture decreases. In Figure 6, the voids of 10–100 mm^3^ are the main part, while voids larger than 100 mm^3^ are sporadically distributed in the mixture. The larger the void volume, the lower the density of the distribution in the asphalt mixture, and the further toward the middle and bottom of the specimen the distribution area aggregates.

To understand the proportion of different sized voids, the extracted voids are analyzed by statistical methods. Considering the quantity and volume of voids, two indicators are used to describe the characteristics of voids: Void volume and void quantity. To cover all the voids in the asphalt mixture, the voids are divided into three categories: smaller than 1 mm^3^, 1–10 mm^3^ and larger than 10 mm^3^. The results are shown in Table 9 and Table 10.

**Figure 7:**
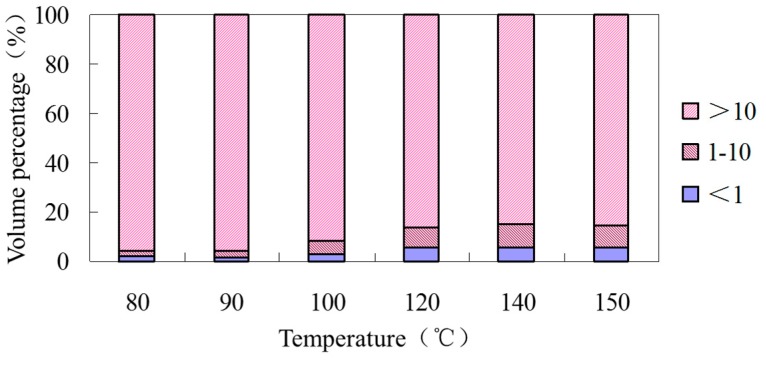
Void distribution of mixtures compacted at different temperatures.

From Table 9 and Table 10, it can be seen that, compared to the hot mix asphalt mixtures mixing at 150 °C, under low-temperature (80–100 °C) mixing conditions, the quantity of small voids (<1 mm^3^), the maximum void volume and the average void volume are larger. When the temperature is higher than 120 °C, there is less difference between the maximum and average void volume. The results show that the void distribution of the warm mix asphalt mixture is close to that of the hot mix asphalt mixture when the temperature is higher than 120 °C, but when the temperature is lower than 120 °C, larger interconnected voids will appear in the warm mix asphalt mixture, resulting in a higher maximum void volume. The volume percentage of different size voids is summarized in Figure 7.

The volume proportions of voids in zeolite asphalt mixture at 120 °C and 140 °C are similar to that of hot mix asphalt mixture, which indicates that the void structures of hot and warm mix asphalt mixtures are basically the same. However, below 100 °C, the proportion of large voids increases, and the small voids decrease correspondingly.

**Figure 8:**
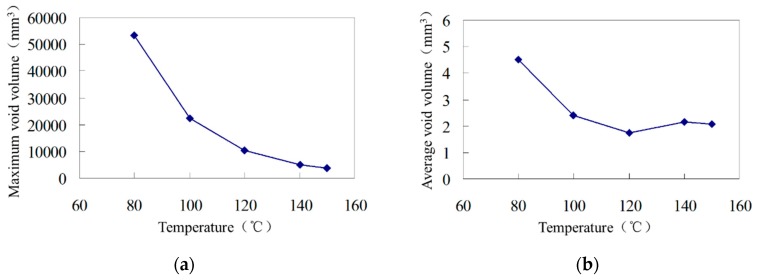
Maximum and average voids of compacted mixtures. (**a**) Maximum void; (**b**) average void.

The average void volume is defined as the total void volume divided by the quantity of voids. The average void volumes of asphalt mixtures at different mixing temperatures are shown in Figure 8. With the increase in temperature, the maximum and average void volumes in the mixture decrease. Therefore, the internal void structure of the asphalt mixture is sensitive to the mixing temperature, and different temperatures lead to different void compositions of the asphalt mixture. Similarly, it can be seen that the maximum and average void volumes can reflect the void characteristics of the mixture. Zeolite asphalt mixture is equivalent to hot mix asphalt mixture at 120 °C and 140 °C, while at temperatures below 120 °C, the maximum and average void volume increase significantly.

It can be seen that the compaction temperature of zeolite asphalt mixture should be maintained at above 120 °C based on the void ratio and void composition information. In this condition, zeolite asphalt mixture has a void ratio and internal void composition close to that of hot mix asphalt mixture. When the temperature is lower than that, the void ratio and average void volume of asphalt mixture increase obviously, and the large void volume, in particular, increases greatly, making it difficult for the mixture to achieve ideal compaction effect.

## 4. Conclusions

This paper proposes an extraction method of voids in zeolite asphalt and asphalt mixture based on the CT scan method; the quantity and volume distributions are also analyzed. The achievements that can be drawn from the results presented in this paper can be summarized as follows.

(1) Micro CT is utilized to obtain the internal structure image of zeolite-modified asphalt and asphalt mixture. Combined with VG Studio MAX 2.2 software, the quantity and volume of the voids in asphalt and asphalt mixture can be extracted.

(2) For the voids in zeolite-modified asphalt at the different mixing temperatures (100 °C and 120 °C), the quantity of voids smaller than 0.1 mm^3^ is larger at 120 °C, but the quantity percentage is lower. This phenomenon shows that with the increase in temperature, there is an obvious evolution trend of smaller void to larger voids. For the void volume distribution, the volume distribution of different sized voids is more uniform at 100 °C. However, for 120 °C, voids larger than 1 mm^3^ account for the vast majority. This illustrates that with the increase in temperature, smaller voids (<1 mm^3^) are merged into larger voids (>1 mm^3^).

(3) For the voids in zeolite-modified asphalt mixture under low temperature (80–100 °C) mixing conditions, the quantity of small voids (<1 mm^3^), the maximum void volume and the average void volume are larger. With the increase in temperature, the maximum and average void volume in the asphalt mixture decrease. Zeolite asphalt mixture is equivalent to hot mix asphalt mixture at 120 °C and 140 °C, while at temperatures below 120 °C, the maximum and average void volume increase significantly, making it difficult for the mixture to achieve ideal compaction effect.

(4) Based on the void distribution in zeolite asphalt binder and zeolite asphalt mixture, it can be seen that temperature has a significant impact on void distribution. In fact, temperature affects the water evaporation, and then influences the tiny foams. Therefore, this paper gives a new method for realizing the void distribution in zeolite asphalt binder and zeolite asphalt mixture. In the future, the effect of the zeolite water content and existence state on the void distribution will be investigated.

## Figures and Tables

**Table 1:** Properties of AH-70 asphalt.

Test Method	Unit	Test Results	Requirements
Penetration (25 °C)	0.1 mm	71.9	60~80
Ductility (15 °C)	cm	>100	≥100
softening point (R&B)	°C	47.0	≥46

**Table 2:** Testing results of coarse aggregates.

Size (mm)	Apparent Relative Density	Water Absorption (%)	Crushing Value (%)	Los Angeles Abrasion (%)
4.75	2.804	0.69	12.3	14.3
9.5	2.844	0.39		
13.2	2.773	0.36		
16	2.801	0.36		
19	2.791	0.33		
26.5	2.795	0.31		
**Requirements**	≥2.60	≤2.0	≤28	≤30

**Table 3:** Apparent density of fine aggregates.

**Size (mm)**	0.075	0.15	0.3	0.6	1.18	2.36
**Apparent Relative Density**	2.791	2.810	2.805	2.823	2.833	2.832
**Requirements**	≥2.50					

**Table 4:** Properties of zeolite.

Apparent Density (g/cm^3^)	Average Diameter (μm)	Maximum Moisture Content (%)
2.297	8.54	17.8

**Table 5:** Gradation of AC-20.

Size (mm)	26.5	19	16	13.2	9.5	4.75	2.36	1.18	0.60	0.30	0.15	0.075
**Design Gradation (%)**	100	95.0	83.0	72.0	57.0	38.0	26.0	18.0	13.0	9.0	6.5	5.0
**Gradation Limitation (%)**	100	90–100	76–90	64–80	50–64	33–43	21–31	13–23	9–17	6–12	4–9	3–7

**Table 6:** Quantity of voids in asphalt.

Temperature (°C)	Voids Quantity				Quantity Percentage (%)			
	<0.1	0.1–0.5	0.5–1	>1	<0.1	0.1–0.5	0.5–1	>1
100	36402	1365	83	44	96.1	3.6	0.2	0.1
120	2076	9	1	7	99.2	0.4	0.1	0.3

**Table 7:** Volume of voids in asphalt.

Temperature (°C)	Voids Ratio (%)	Volume Percentage (%)				Void Radius (mm)		
		<0.1	0.1–0.5	0.5–1	>1	Max	Min	Average
100	1.83	64.0	22.0	4.8	9.2	1.30	0.13	0.19
120	13.9	1.05	0.04	0.03	98.88	3.97	0.13	1.19

**Table 8:** Void ratio of zeolite asphalt mixture (%).

Compaction Temperature (°C)	150	140	130	120	110	100	90	80
**Void Ratio (%)**	4.21	4.07	4.34	4.59	5.73	7.43	11.13	13.16

**Table 9:** Quantity and volume of voids in asphalt mixtures.

Temperature (°C)	Void Quantity			Void Volume (mm^3^)	
	<1 mm^3^	1–10 mm^3^	>10 mm^3^	Maximum	Average
80	12161	457	44	53270.6	4.5
90	7710	488	54	50822.5	6.7
100	13813	660	96	22390.8	2.4
120	11833	683	79	10326.3	1.7
140	8154	676	129	5065.8	2.1
150	8888	646	99	3832.2	2.1

**Table 10:** Volume proportion of voids in asphalt mixtures (%).

Temperature (°C)	<1 mm^3^	1–10 mm^3^	>10 mm^3^
80	1.9	2.0	96.1
90	1.6	2.4	96.0
100	3.0	5.2	91.8
120	5.8	8.3	85.9
140	5.5	9.7	84.8
150	5.6	8.6	85.8
